# An Omnibus Test for Detecting Multiple Phenotype Associations Based on GWAS Summary Level Data

**DOI:** 10.3389/fgene.2021.644419

**Published:** 2021-03-17

**Authors:** Wei Liu, Yunshan Guo, Zhonghua Liu

**Affiliations:** Department of Statistics and Actuarial Science, The University of Hong Kong, Hong Kong, China

**Keywords:** multiple phenotypes, summary statistics, the generalized higher criticism, the generalized Berk-Jones test, the aggregated Cauchy association test

## Abstract

Abundant Genome-wide association study (GWAS) findings have reflected the sharing of genetic variants among multiple phenotypes. Exploring the association between genetic variants and multiple traits can provide novel insights into the biological mechanism of complex human traits. In this article, we proposed to apply the generalized Berk-Jones (GBJ) test and the generalized higher criticism (GHC) test to identify the genetic variants that affect multiple traits based on GWAS summary statistics. To be more robust to different gene-multiple traits association patterns across the whole genome, we proposed an omnibus test (OMNI) by using the aggregated Cauchy association test. We conducted extensive simulation studies to investigate the type one error rates and compare the powers of the proposed tests (i.e., the GBJ, GHC and OMNI tests) and the existing tests (i.e., the minimum of the *p*-values (MinP) and the cross-phenotype association test (CPASSOC) in a wide range of simulation settings. We found that all of these methods could control the type one error rates well and the proposed OMNI test has robust power. We applied those methods to the summary statistics dataset from Global Lipids Genetics Consortium and identified 19 new genetic variants that were missed by the original single trait association analysis.

## Introduction

Genome-wide association studies (GWASs) have identified thousands of genetic variants or single nucleotide polymorphisms (SNPs) that are associated with hundreds of complex human traits (Solovieff et al., 2013). The abundance of GWASs findings provides novel insights into the genetic architecture of complex human traits and suggests the existence of sharing of SNPs among multiple traits (Luo et al., 2020). Therefore, there is an increasing interest in exploring powerful statistical methods to detect the association between a single SNP and multiple traits (Liu and Lin, 2018). The existing methods can be broadly classified into multivariate approaches and univariate approaches (Solovieff et al., 2013). Multivariate approaches analyze all of the interesting traits in a unified framework (Solovieff et al., 2013; Zhu et al., 2015). However, they require to pool the individual-level phenotype and genotype data, which are always difficult to access (Pasaniuc and Price, 2017). In contrast, univariate approaches try to aggregate the GWAS results of each trait to jointly analyze multiple traits while accounting for the between-trait correlation (Liu and Lin, 2018). The most attractive advantage of univariate approaches is that they can be implemented by existing GWAS summary statistics, which contain rich information and are also easier to access than individual-level data. The minimum of the *p*-values (MinP) (Conneely and Michael, 2007) of multiple traits is one of the most classical univariant methods that accounting for the correlation structures among multiple phenotypes. It has been demonstrated to be powerful when signals are extremely sparse, that means a SNP only affects a very small number of multiple traits (Liu and Lin, 2018). However, it may lose power when the signals become dense. Later, Zhu et al. (2015) proposed two cross-phenotype association tests (CPASSOC) to integrate the evidence of multiple phenotypes. However, these two tests strongly depend on the assumption of homogeneous or heterogeneous effect. If the assumption is violated, they will lose power. In addition, the *P*-value of these tests need to be calculated by Monte-Carlo simulations, which are computationally intensive especially for large-scale genome data (Liu and Lin, 2018). It is hence of substantial interest to develop robust and computationally efficient statistical tests to jointly analyze multiple traits based on the univariate GWAS summary statistics.

The higher criticism (HC) (Donoho and Jin, 2004) and the Berk-Jones (BJ) (Berk and Jones, 1979) test are two efficient ways to aggregate the sparse and weak signals into a stronger one when signals are independent. To account for the correlation among marginal test statistics, the generalized higher criticism (GHC) test (Barnett et al., 2017) has been proposed and used for SNP-set analysis. The GHC test neither requires any transformation of the original test statistics nor simulation-based procedure to obtain the *P*-value. Several previous studies have shown that the GHC test has good performance under extreme sparsity settings while might lose power under moderate sparsity settings. To adapt the case of weak and moderately sparsity signals, Sun and Lin developed the generalized Berk-Jones (GBJ) test (Sun and Lin, 2020) also in the SNP-set analysis context. The GBJ test is an extension of the BJ test while considering the correlation structure of signals. Besides, they provided a more computationally efficient analytic *P*-value calculation procedure. Both the GHC and GBJ tests are originally designed to test for association between a SNP-set and an outcome, and they haven’t been adapted for multi-trait analysis. Since the power of multi-trait analysis depends on the correlation structure, the signal directions and the signal sparsity which typically varies with genetic variants across the whole genome, therefore, a more robust omnibus testing procedure is deserved. Recently, Liu et al. proposed an attractive *P*-value combination method, i.e., the aggregated Cauchy association test (ACAT) (Liu et al., 2019). The ACAT is not only robust to the correlation structure between combined *P*-values but is also computationally efficient.

In this article, we presented powerful procedures to jointly analyze multiple traits based on GWAS summary statistics. First, we adapted the GBJ and GHC tests originally developed for SNP-set association studies to analyze multiple traits. We replaced the linkage disequilibrium (LD) matrix among SNPs with the correlation matrix among multiple traits. Second, we proposed a more robust omnibus (OMNI) test to combine the *P*-values of the GBJ, GHC and MinP tests by the ACAT. We investigated the type one error rates of the proposed tests (i.e., The GBJ, GHC and OMNI tests) and compared their statistical powers with the existing tests (i.e., the MinP and CPASSOC tests) by conducting extensive simulations. Through analyzing three lipid traits, i.e., high-density lipoprotein (HDL), low-density lipoprotein (LDL) and triglyceride (TG) from the global lipids genetics consortium (GLGC) (Teslovich et al., 2010), we identified 19 new SNPs that were ignored by the original univariate GWAS.

## Materials and Methods

Consider *N* subjects and *K* correlated phenotypes **Y** = (*Y*_1_,⋯,*Y*_*K*_)^*T*^, where *T* denotes the transpose of a vector or matrix. Traditional GWAS is used to performing univariate phenotype analysis by analyzing each of the *K* phenotypes and a given SNP separately, which generate *K* marginal test statistics (Liu and Lin, 2018). Suppose **Z** = (*Z*_1_,…,*Z*_*K*_)^*T*^ (*k* = 1,…,*K*) is a vector of Z scores with each element of **Z** obtained from large-scale GWASs. Under the null hypothesis *H*_*0*_, **Z** asymptotically follows a multivariate normal distribution with mean μ and covariance matrix **Σ** (Zhu et al., 2015; Liu and Lin, 2018). The covariance matrix **Σ** can be accurately estimated by the sample correlation matrix across all the independent SNPs after LD pruning under *H*_*0*_ (Liu and Lin, 2018). Since this estimation procedure is valid if the GWAS summary statistics are obtained from one cohort, or multiple cohorts with possible overlapping subjects or phenotype-specific meta-analysis (Liu and Lin, 2018), our *K* phenotypes allow being measured on the same or the different study subjects. When jointly analyzing multiple traits, we are interested in exploring whether a given SNP is associated with these *K* correlated phenotypes, that is to test the hypothesis *H*_0_:*μ* = 0 versus *H*_*a*_:*μ*≠0 (i.e., at least one element of μ is not equal to zero). Since there may exist sparse signal (Liu and Lin, 2018), that is only a small set of non-zero values in μ under *H*_*a*_, we first adapted the GBJ and GHC tests to detect these weak and sparse signals.

### The Generalized Higher Criticism Test

The HC test is an attractive method to detect the sparse signals in high-dimensional data when test statistics are independent and the number of parameters is very large (Barnett and Lin, 2014). The GHC test statistic is an extension of the HC test and allows for arbitrary correlation structures. This test has been used to account for the LD structure among SNPs when performing gene-based genome-wide association analyses. However, to jointly analyze multiple phenotypes, we account for the between-phenotype correlation structure **Σ** rather than the LD structure because we conduct association analysis for each SNP individually. Therefore, the definition of the GHC test statistic for combining information over *K* marginal test statistics can be written as

$$\text{GHC}=\begin{array}{c} s\,u\,p\\ t\geq t_{0} \end{array}\,\left\{\frac{S\,\left(t\right)-2\,K\,\bar{\Phi }\,\left(t\right)}{\sqrt{\hat{v\,a\,r}\,\left(S\,\left(t\right)\right)}}\right\},$$

where $\bar{\Phi }\,\left(t\right)=1-\Phi \,\left(t\right)$ is the survival function of a normal distribution, $S\,\left(t\right)=\sum_{k=1}^{K}1_{\left\{|Z_{k}|\geq t\right\}}$, $\hat{v\,a\,r}\,\left(S\,\left(t\right)\right)$ is the estimated variance of *S*(*t*) that can be obtained by **Σ** as demonstrated by Barnett et al. (2017). The *P*-value of the GHC test can be calculated analytically, refer to Barnett et al. (2017) for more details.

### The Generalized Berk-Jones Test

The GBJ test is derived from a class of tests that originally developed to test for the association between a SNP-set and an outcome (Sun and Lin, 2020), while accounting for LD structures among SNPs. Here, we also use the between-phenotype correlation structure **Σ** to replace the LD structure among SNPs. Then, the GBJ test for testing *K* multiple phenotypes can be defined as the maximum of a set of likelihood ration type tests, that is:

$$=\max_{1\leq k\leq K/2}\log\left[\frac{\text{Pr}\left\{S\left(|Z{|}_{\left(K-k+1\right)}\right)=k|E\left(\text{Z}\right)={\hat{\mu }}_{k,K}\cdot {\text{J}}_{K},\text{cov}\left(\text{Z}\right)=\Sigma \right\}}{\text{Pr}\left\{S\left(|Z{|}_{\left(K-k+1\right)}\right)=k|E\left(\text{Z}\right)=0\cdot {\text{J}}_{K},\text{cov}\left(\text{Z}\right)=\Sigma \right\}}\right]\cdot I\left\{2\Phi \left(|Z{|}_{\left(k-K+1\right)}\right)<\frac{k}{K}\right\},$$

where **I** is an indicator function, **J**_*K*_ is a *K*×1 unit vector and ${\hat{\mu }}_{k,K}>0$ solves the following equation

$$k/K=1-\left\{\Phi \,\left({\left|Z\right|}_{\left(K-k+1\right)}-{\hat{\mu }}_{k,K}\right)-\Phi \,\left(-{\left|Z\right|}_{\left(K-k+1\right)}-{\hat{\mu }}_{k,K}\right)\right\}.$$

When **Σ** = **I**, the GBJ test reduces to the BJ test and *S*(*t*) follows a binomial distribution. When **Σ**≠**I**, the distribution of *S*(⋅) can be approximated by an extended Beta-Binomial distribution (Prentice, 1986), so that the analytical *P*-value can be calculated efficiently, refer to Sun et al. for more detail (Sun and Lin, 2020). This *P*-value calculation strategy can also be generalized to other supremum-based tests, such as the HC and GHC tests. Sun and Lin (2020) constructed an R package named GBJ, that can provide both the test statistics and the *P*-values of the GBJ, GHC and MinP tests^1^.

### The Omnibus Test

As demonstrated by Sun and Lin (2020), the GBJ test is better when signals are moderately spare, while the GHC and MinP tests have better performance when the signals were extremely sparse. We further proposed an omnibus test based on the ACAT method that is more robust to different degrees of sparsity and correlation structures. We define our omnibus test as

$$\text{OMNI}=\frac{1}{3}\,\tan\left\{\left(0.5-p_{\text{GBJ}}\right)\,\pi \right\}+\frac{1}{3}\,\tan\left\{\left(0.5-p_{\text{GHC}}\right)\,\pi \right\}+\frac{1}{3}\,\tan\left\{\left(0.5-p_{\text{MinP}}\right)\,\pi \right\},$$

where*p*_*GBJ*_, *p*_*GHC*_ and *p*_*MinP*_ are the *P*-values of the GBJ, GHC and MinP tests. Since *p*_*GBJ*_, *p*_*GHC*_ and *p*_*MinP*_ are calculated under the null distribution, the transformation $\frac{1}{3}\,\tan\left\{\left(0.5-p\right)\,\pi \right\}$ follows Cauchy distribution (Liu et al., 2019). Therefore, the OMNI test is approximately following a Cauchy distribution with a location parameter 0 and a scale parameter 1. The *P*-value of the OMNI test can be calculated by

$$p_{\text{OMNI}}\approx \frac{1}{2}-\frac{a\,r\,c\,t\,a\,n\,\left(\text{OMNI}\right)}{\pi }.$$

The program from https://rdrr.io/github/xihaoli/STAAR/src/R/CCT.R can be used to directly implement the ACAT method. We also developed an R package for the above proposed tests^2^.

## Simulation Study

### Type One Error Rates

We conducted simulation studies to investigate the type one error rates of the GBJ, GHC, MinP and OMNI tests at significance levels *α* = 0.05, 0.01, 10^−3^ and 10^−4^ respectively. We set the number of traits *K* equal to 2, 4 and 10. For a given *K*, the correlation matrix **Σ** of interested traits was exchangeable with the correlation coefficient *ρ* = 0.1, 0.3, 0.5. Based on these, we generated 10^3^, 10^4^, 10^5^, and 10^6^ summary statistics by a multivariate normal distribution with mean ***0*** and covariance matrix **Σ**. Then we calculated the *P*-values of the GBJ, GHC, MinP and OMNI tests for each of the above settings. The type one error rates for each test were calculated as the proportion of *P*-values less than the significance level. The results were summarized in Table 1, which showed that the type one error rates could be well controlled by all of these tests at different significant levels in multiple-trait analysis settings.

**TABLE 1:** Type I error rates of GBJ, GHC, MinP and OMNI tests at significance level of *α* = 0.05, 0.01, 10^−3^ and10^−4^ based on 1×10^3^, 1×10^4^, 1×10^5^ and 1×10^6^ replications respectively. The correlation matrix is exchangeable with off-diagonal element equal to ρ.

ρ	α	*K = 2*				*K = 4*				*K = 10*			
		GBJ	GHC	MinP	OMNI	GBJ	GHC	MinP	OMNI	GBJ	GHC	MinP	OMNI
0.1	0.05	0.047	0.048	0.047	0.047	0.052	0.050	0.058	0.051	0.046	0.052	0.063	0.055
0.3		0.051	0.054	0.051	0.051	0.047	0.044	0.046	0.045	0.033	0.035	0.042	0.037
0.5		0.052	0.047	0.052	0.048	0.056	0.054	0.064	0.057	0.032	0.033	0.050	0.038
0.1	0.01	0.010	0.010	0.010	0.010	0.009	0.009	0.009	0.010	0.012	0.012	0.012	0.011
0.3		0.011	0.011	0.011	0.011	0.010	0.010	0.010	0.011	0.010	0.010	0.010	0.011
0.5		0.011	0.010	0.011	0.010	0.008	0.008	0.009	0.008	0.006	0.007	0.010	0.008
0.1	1×10^−3^	7.8×10^−4^	7.9×10^−4^	7.8×10^−4^	7.8×10^−4^	9.6×10^−4^	9.7×10^−4^	9.5×10^−4^	1.0×10^−3^	1.2×10^−3^	9.6×10^−4^	9.4×10^−4^	1.1×10^−3^
0.3		9.1×10^−4^	9.0×10^−4^	9.1×10^−4^	9.1×10^−4^	1.0×10^−3^	1.0×10^−3^	1.0×10^−3^	1.0×10^−3^	1.1×10^−3^	1.1×10^−3^	1.1×10^−3^	1.2×10^−3^
0.5		1.1×10^−3^	1.1×10^−3^	1.1×10^−3^	1.1×10^−3^	8.9×10^−4^	9.8×10^−4^	1.0×10^−3^	9.7×10^−4^	7.6×10^−4^	9.8×10^−4^	1.0×10^−3^	9.8×10^−4^
0.1	1×10^−4^	1.0×10^−4^	1.2×10^−4^	9.4×10^−5^	9.6×10^−5^	1.1×10^−4^	1.2×10^−4^	1.2×10^−4^	1.2×10^−4^	1.3×10^−4^	9.8×10^−5^	9.8×10^−5^	1.2×10^−4^
0.3		9.3×10^−5^	1.0×10^−4^	1.0×10^−4^	1.0×10^−4^	1.0×10^−4^	1.1×10^−4^	1.1×10^−4^	1.2×10^−4^	1.3×10^−4^	1.0×10^−4^	1.0×10^−4^	1.2×10^−4^
0.5		9.6×10^−5^	1.1×10^−4^	1.1×10^−4^	1.1×10^−4^	9.0×10^−5^	9.3×10^−5^	9.3×10^−5^	9.9×10^−5^	1.1×10^−4^	8.1×10^−5^	8.4×10^−5^	9.9×10^−5^

### Power

We further compared the empirical powers of the proposed tests (i.e., the GBJ, GHC and OMNI tests) with the existing MinP and CPASSOC tests. The powers were calculated by the proportion of *P*-values less than the significant level *α* = 0.05. In particular, we account for the following factors: signal direction, signal sparsity and the correlation structure among multiple traits. First, we considered the number of traits *K = 2* with *μ* = (2,2)^*T*^ and *μ* = (2,−2)^*T*^ respectively to illustrate the effect of the signal direction on the power of multi-trait analysis. For each μ, we set the correlation coefficient *ρ* = 0.1, 0.3, 0.5 and 0.8 to investigate how the correlation structure affects the power. Second, we considered *K = 3* and two correlation structures **Σ**_1_ and **Σ**_2_, i.e.,

$$\Sigma_{1}=\left[\begin{array}{ccc} 1.00 & 0.30 & 0.30\\ 0.30 & 1.00 & 0.30\\ 0.50 & 0.30 & 1.00 \end{array}\right],\Sigma_{2}=\left[\begin{array}{ccc} 1.00 & -0.08 & -0.42\\ -0.08 & 1.00 & 0.27\\ -0.42 & 0.27 & 1.00 \end{array}\right].$$

Here, **Σ**_2_ is estimated from the real summary statistics of three lipid traits (i.e., HDL, LDL and TG). We investigated the impact of the location of the heterogeneity effect on power. The details of the settings for each correlation structure were listed in Table 2. Third, we consider *K = 10* and *K = 20* to investigate the effect of signal sparsity. For both *K = 10* and *K = 20*, we set the correlation coefficient *ρ* = 0.3 and allowed 1, 3, 6 and 9 traits among them with a mean value of 2. We generated 1,000 random samples based on a multivariate normal distribution with the above different mean μ and covariance matrix **Σ**. The *P*-values are then calculated by the GBJ, GHC, OMNI, MinP and CPASSOC tests, respectively.

**TABLE 2:** Estimated power of the GBJ, GHC, MinP, OMNI and CPASSOC tests when *K = 3* at significance level of *α* = 0.05 based on 1×10^3^ replications for each setting.

Σ	*μ*^*T*^	GBJ	GHC	MinP	OMNI	CPASSOC
**Σ**_1_	(**−2**, 2, 2)	0.792	0.793	0.749	0.802	0.146
	(2, **−2**, 2)	0.832	0.823	0.778	0.827	0.153
	(2, 2, **−2**)	0.818	0.799	0.749	0.808	0.150
**Σ**_2_	(**−2**, 2, 2)	0.719	0.720	0.670	0.728	0.094
	(2, **−2**, 2)	0.829	0.811	0.757	0.821	0.686
	(2, 2, **−2**)	0.774	0.757	0.726	0.768	0.164
**Σ**_1_	(**−**2, **−**2, **2**)	0.794	0.789	0.755	0.799	0.140
	(**−**2, **2**, **−**2)	0.831	0.821	0.779	0.829	0.168
	(**2**, **−**2, **−**2)	0.810	0.803	0.754	0.808	0.144
**Σ**_2_	(**−**2, **−**2, **2**)	0.779	0.775	0.719	0.778	0.149
	(**−**2, **2**, **−**2)	0.869	0.841	0.798	0.854	0.726
	(**2**, **−**2, **−**2)	0.724	0.714	0.675	0.719	0.087

**FIGURE 1:**
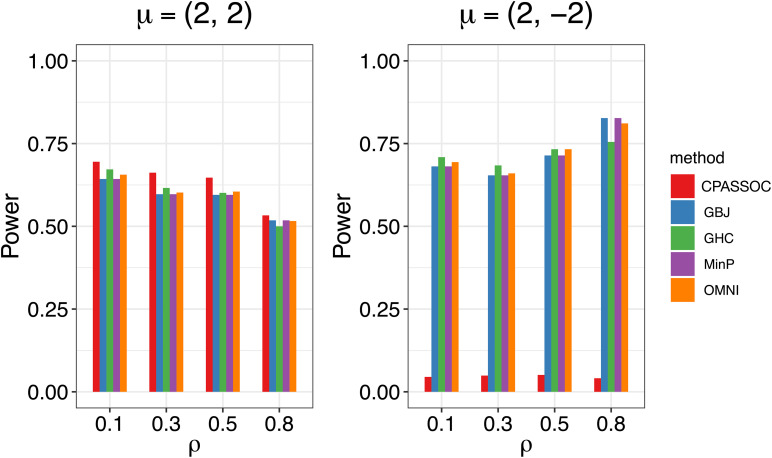
Estimated powers of the GBJ, GHC, OMNI, MinP and CPASSOC tests when *K*= 2 based on 1,000 replications at the significant level *α*=0.05.

**FIGURE 2:**
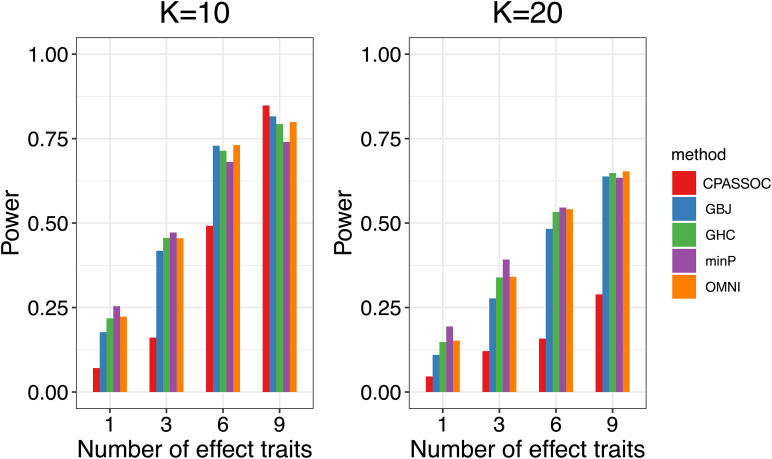
Estimated powers of the GBJ, GHC, OMNI, MinP and CPASSOC tests when *ρ*=0.3 based on 1,000 replications at the significant level *α*=0.05.

Figure 1 shows the estimated powers of the GBJ, GHC, OMNI, MinP and CPASSOC tests when *K = 2*. Regardless of the correlation structures, the CPASSOC test has the largest power for the homogeneous effect *μ* = (2,2)^*T*^. However, it decreased less than 0.1 for the heterogeneous effect *μ* = (2,−2)^*T*^, which is consistent with Zhu et al.’s simulation (Zhu et al., 2015). In contrast, our proposed tests (i.e., the GBJ, GHC and OMNI tests) and the MinP test are robust to the heterogeneous effect *μ* = (2,−2)^*T*^. With the correlation increasing from 0.1 to 0.8, the powers of all the tests decrease for *μ* = (2,2)^*T*^, while increasing for *μ* = (2,−2)^*T*^. In addition, the GHC test is more powerful than the GBJ and MinP tests with smaller ρ, while less powerful with larger ρ. The performance of the GBJ and MinP tests are very similar when *K = 2*. They have the largest power for heterogeneous effect *μ* = (2,−2)^*T*^ with *ρ* = 0.8. The OMNI test has a moderate performance among all of the settings. The estimated powers of the GBJ, GHC, OMNI, MinP and CPASSOC tests when *K = 3* were summarized in Table 2. Except for the CPASSOC test that less powerful for the heterogeneous effect, all other tests perform well under both **Σ**_1_ and **Σ**_2_. The GBJ test always has the best performance, closely followed by the OMNI and GHC tests. The performance of the MinP test is worse than all of our proposed tests when *K = 3*. Interestingly, all the tests have the largest power for *μ* = (2,−2,2)^*T*^, while lowest power for *μ* = (−2,2,2)^*T*^. Figure 2 shows the estimated powers of the GBJ, GHC, OMNI, MinP and CPASSOC tests when *K = 10* and *K = 20* allowing 1, 3, 6 and 9 traits with non-zero effect. As expected, the GBJ test almost outperforms other tests when the signals are moderately sparse while has the lowest power when the signals are extremely sparse. On the contrary, the MinP test provides the best power performance under extreme sparse while losing power as the signals becoming dense. The performance of the GHC test approximates the MinP test when the number of affected traits is very small, but it is less sensitive to the signal sparsity than the MinP test. The CPASSOC test performs poorly when the signals are sparse, while becomes better when nearly all the traits with non-zero effect. As shown in Table 2, Figures 1 and 2, there is no single test that can outperform others among all the settings. In contrast, the OMNI test is robust to the signal directions, the correlation structures and the degrees of sparsity.

## Real Data Analysis

We now illustrate the proposed methods using the summary statistics of three lipid traits (i.e., HDL, LDL and TG) provided by the GCLC (Teslovich et al., 2010) from http://csg.sph.umich.edu/willer/public/lipids2010/. We began our real data analysis with a total of 2,691,421 SNPs that were shared by these three traits. Based on these SNPs, we calculated the sample correlation matrix **Σ**_2_ of Z-scores of HLD, LDL and TG using the same method as in Liu and Lin (2018). We used the GBJ, GHC, OMNI, MinP and CPASSOC tests to calculate the *P*-values and obtained 68 new SNPs (missed by single-trait analysis) that reached the genome-wide significance level (5×10^−8^) according to the *P*-values of the OMNI test. Here, for comparison, we used the same genome-wide significance level as in the original single-trait analysis paper (Teslovich et al., 2010). To avoid dependent SNPs, we conducted LD pruning with threshold *r*^2^< 0.01 within the 500kb region (Liu and Lin, 2018). Finally, we found 19 independent new SNPs that were missed by all of the three original GWAS. The detail information of these SNPs was listed in Table 3. For example, the most significant SNP found by the ONMI test is rs7307053 (*p* = 3.05×10^−10^), which was also identified by the GBJ test with *p* = 1.02×10^−10^. The CPASSOC test identified 5 new SNPs, which provided the most significant SNP rs11823918 (*p* = 1.55×10^−20^). The GHC and MinP tests did not found new SNPs. The genomic inflation factor of these five tests was very close to 1.0 (0.98–1.10). Overall, the real data analysis results further demonstrated that the proposed OMNI test is a useful method to detect novel SNPs for multiple-trait analysis.

**TABLE 3:** Application of the GBJ, GHC, OMNI, MinP and CPASSOC tests to the GCLC dataset with *P*-values of the OMNI test less than the GWAS significant level 5×10^−8^.

SNP	Chr	*p*_*HDL*_	*p*_*LDL*_	*p*_*TG*_	GBJ	GHC	MinP	OMNI	CPASSOC
rs7307053	12	5.53×10^−8^	1.94×10^−3^	1.01×10^−7^	1.02×10^−10^	7.62×10^−8^	1.66×10^−7^	3.05×10^−10^	0.637
rs3890384	19	0.386	7.06×10^−8^	2.07×10^−7^	2.84×10^−10^	1.55×10^−7^	2.11×10^−7^	8.49×10^−10^	3.33×10^−11^
rs330095	8	7.67×10^−8^	2.19×10^−7^	0.228	3.07×10^−10^	1.64×10^−7^	2.30×10^−7^	9.17×10^−10^	2.69×10^−8^
rs6993714	8	3.02×10^−7^	0.023	1.80×10^−7^	4.90×10^−10^	2.27×10^−7^	5.40×10^−7^	1.47×10^−9^	0.711
rs3918241	20	3.71×10^−7^	0.799	2.62×10^−7^	6.59×10^−10^	2.79×10^−7^	7.85×10^−7^	1.97×10^−9^	0.585
rs968567	11	8.32×10^−5^	3.87×10^−7^	4.96×10^−7^	9.98×10^−10^	3.72×10^−7^	1.16×10^−6^	2.98×10^−9^	0.176
rs11823918	11	1.32×10^−7^	0.011	5.56×10^−7^	1.18×10^−9^	3.96×10^−7^	3.96×10^−7^	3.51×10^−9^	1.55×10^−20^
rs1112073	6	0.242	5.77×10^−7^	4.38×10^−7^	1.24×10^−9^	4.33×10^−7^	1.31×10^−6^	3.71×10^−9^	3.78×10^−6^
rs11754548	6	6.56×10^−7^	2.91×10^−7^	0.593	1.49×10^−9^	4.92×10^−7^	8.74×10^−7^	4.46×10^−9^	1.14 ×10^−8^
rs13432797	2	6.74×10^−7^	1.68×10^−4^	5.16×10^−8^	1.55×10^−9^	1.55×10^−7^	1.55×10^−7^	4.56×10^−9^	0.230
rs2943633	2	9.43×10^−7^	0.139	3.71×10^−7^	2.52×10^−9^	7.09×10^−7^	1.11×10^−6^	7.53×10^−9^	0.863
rs489693	18	1.49×10^−6^	0.099	4.81×10^−7^	4.86×10^−9^	1.12×10^−6^	1.44×10^−6^	1.45×10^−8^	0.371
rs653178	12	1.59×10^−6^	8.60×10^−8^	0.203	5.34×10^−9^	2.58×10^−7^	2.58×10^−7^	1.54×10^−8^	3.93×10^−7^
rs636202	6	6.48×10^−8^	0.011	1.64×10^−6^	5.62×10^−9^	1.95×10^−7^	1.94×10^−7^	1.59×10^−8^	0.917
rs10516787	4	2.11×10^−6^	0.043	1.80×10^−6^	8.10×10^−9^	1.59×10^−6^	5.41×10^−6^	2.41×10^−8^	0.788
rs157582	19	2.49 ×10^−3^	2.24×10^−6^	7.99×10^−8^	8.76×10^−9^	2.40×10^−7^	2.40×10^−7^	2.45×10^−8^	1.55×10^−3^
rs1240777	11	2.19×10^−6^	0.3751	1.36×10^−6^	8.52×10^−9^	1.65×10^−6^	4.09×10^−6^	2.54×10^−8^	0.932
rs511154	3	2.69×10^−6^	0.191	1.01×10^−7^	1.15×10^−8^	3.03×10^−7^	3.03×10^−7^	3.20×10^−8^	0.632
rs10790519	11	5.62×10^−8^	3.98×10^−6^	0.729	2.03×10^−8^	1.68×10^−7^	1.68×10^−7^	4.90×10^−8^	4.24×10^−11^

## Discussion

As there is increasing evidence suggesting the sharing of genetic background among multiple traits, it is of great interest to develop robust and powerful statistical methods to detect the association between a single SNP and multiple traits. In this article, we proposed to use the GBJ and GHC tests for multi-traits analysis based on GWAS summary statistics. Since no single test can have superior performance among different situations, we further proposed a more robust omnibus test using the ACAT method. Simulation studies showed that the proposed OMNI test has a robust performance across different settings. Through analyzing the lipids GWAS data of three traits (i.e., HDL, LDL and TG), our proposed OMNI test identified new SNPs that were missed by original single-trait GWAS analysis.

Our methods have several advantages. First, our methods are based on the GWAS summary statistics that are easier to access than individual level data for multiple-trait analysis. Second, we adapted two powerful methods (i.e., the GBJ and GHC tests) originally developed for SNP-set association studies to conduct multi-trait analysis while accounting for the correlation structures. Third, we propose an omnibus test that used the recently developed computationally efficient and accurate ACAT method that can provide robust performance over different degrees of association signal sparsity. In summary, all of these tests can be applied to detect SNPs that associated with multiple traits when there exist weak and sparse signals. For future work, we will try to extend our work to test the associations between multiple traits and a set of SNPs which requiring considering both LD among SNPs and the correlation structure among multiple traits as also mentioned by Liu et al. (2020), even including rare variants in sequencing association studies.

## Data Availability Statement

Publicly available datasets were analyzed in this study. This data can be found here: http://csg.sph.umich.edu/willer/public/lipids2010/.

## Author Contributions

ZL initiated and developed the study. WL conducted the simulation study and performed the real data analysis, completed the manuscript writing. YG developed the R package. All authors contributed to the article and approved the submitted version.

## Conflict of Interest

The authors declare that the research was conducted in the absence of any commercial or financial relationships that could be construed as a potential conflict of interest.

## Footnotes

1 https://cran.r-project.org/web/packages/GBJ/index.html

2 https://github.com/Vivian-Liu-Wei64/Onmi_Multi
